# Reliability of meta-analyses in ecology and evolution: (mostly) good news from a case study on sexual signals

**DOI:** 10.1098/rspb.2024.2782

**Published:** 2025-05-21

**Authors:** Pietro Pollo, Malgorzata Lagisz, Renato C. Macedo-Rego, Ayumi Mizuno, Yefeng Yang, Shinichi Nakagawa

**Affiliations:** ^1^ Evolution & Ecology Research Centre, School of Biological, Earth & Environmental Sciences, University of New South Wales, Kensington, New South Wales, Australia; ^2^ Departamento de Biologia Geral, Universidade Federal de Viçosa, Viçosa, Minas Gerais, Brazil; ^3^ Department of Biological Sciences, University of Alberta, Edmonton, Alberta, Canada

**Keywords:** data integrity, methodological accuracy, open science, research synthesis, sexual selection

## Abstract

Meta-analyses are powerful synthesis tools that are popular in ecology and evolution owing to the rapidly growing literature of this field. Although the usefulness of meta-analyses depends on their reliability, such as the precision of individual and mean effect sizes, attempts to reproduce meta-analyses’ results remain rare in ecology and evolution. Here, we assess the reliability of 41 meta-analyses on sexual signals by evaluating the reproducibility and replicability of their results. We attempted to: (i) reproduce meta-analyses’ mean effect sizes using the datasets they provided; (ii) reproduce meta-analyses’ effect sizes by re-extracting 5703 effect sizes from 246 primary studies they used as sources; (iii) assess the extent of relevant data missed by original meta-analyses; and (iv) replicate meta-analyses’ mean effect sizes after incorporating re-extracted and relevant missing data. We found many discrepancies between meta-analyses’ reported results and those generated by our analyses for all reproducibility and replicability attempts. Nonetheless, we argue that the meta-analyses we evaluated are largely reproducible and replicable because the differences we found were small in magnitude, leaving the original interpretation of these meta-analyses’ results unchanged. Still, we highlight issues we observed in these meta-analyses that affected their reliability, providing recommendations to ameliorate them.

## 1. Introduction

The literature of ecology and evolution, like of other fields of study, is expanding rapidly [1,2]. Consequently, synthesizing this growing body of work becomes increasingly necessary to identify patterns across individual studies. Meta-analyses, which are perceived as the gold standard for evidence synthesis, can contribute to this endeavour because they aim to detect and retrieve all relevant studies on a given topic, extract data from these studies and quantify an average effect of interest using the extracted data [3,4]. Nonetheless, many challenges arise during the execution of meta-analytical studies, requiring researchers to be vigilant to ensure their reliability.

Foremost, meta-analyses need to be transparent, which means that they must provide details on decisions and resources regarding all steps of their implementation (e.g. searches, screening, data extraction and analysis code). Yet, recent appraisals of the secondary literature in ecology and evolution show that meta-analyses in this field are often poorly transparent [5–7]. Reporting guidelines, such as MOOSE [8] and PRISMA [6,9], were developed to address transparency issues in evidence syntheses. Researchers who adhere to these guidelines can thus enhance the quality of their meta-analyses. Still, transparency represents only the first step for high-quality syntheses as providing information does not guarantee its reliability. For instance, researchers can make mistakes when describing their methods, provide faulty code for their analyses or even falsify data, impairing the reproducibility and replicability of their findings. Therefore, it is imperative to evaluate studies beyond transparency.

Meta-analyses possess the advantage of having sources of information that are readily accessible (i.e. individual studies from which data are extracted). This means that meta-analyses can be more easily evaluated than other approaches regarding reproducibility and replicability aspects. More specifically, meta-analytical studies that report their decision criteria (i.e. studies and data considered valid for inclusion) and their effect sizes with information on their sources (i.e. studies they were extracted from) should allow evaluations regarding the reproducibility of their dataset and their results. However, to our knowledge, only computational reproducibility of meta-analyses (i.e. reproducibility of their code) has been examined in ecology and evolution [10]. Although meta-analyses with similar goals conducted by independent groups may represent examples of quasi-replications in ecology and evolution (e.g. [11] and [12]; [13] and [14]), reproducibility attempts of data extraction and general results have only been conducted in other fields of study (but see [15]; e.g. medicine [16]; psychology [17]). Furthermore, other elements connected to the reliability of meta-analyses, such as their efficacy in detecting relevant studies, remain virtually unexplored.

Here, using 41 meta-analyses related to sexual signals [18–58], we conduct, to our knowledge, the largest reproducibility and replicability effort for meta-analyses ever done in terms of number of re-extracted primary studies and effect sizes (5703 data points from 246 primary studies, representing almost a fifth of all primary studies in our dataset). We evaluate multiple reliability aspects related to distinct implementation stages of these meta-analyses (electronic supplementary material, figure S1). First, we re-analyse the dataset provided by these meta-analyses, comparing the mean effect sizes we obtained with those reported in them. Second, we extract data from their original sources (i.e. individual studies) and compare these extracted data points with the ones reported in these meta-analyses. Third, we assess how many data points from the original sources that we verified should have been extracted and included in these meta-analyses’ datasets but were not (i.e. ‘missed’ data). Fourth, because these meta-analyses ask similar questions involving sexual signals, we estimate the minimum number of studies that contained relevant data but were not listed as sources in these meta-analyses (i.e. undetected studies). Fifth, we assess the extent that the results of these meta-analyses change when re-extracted data (along with missed data and undetected studies) is analysed in place of originally reported data.

## 2. Methods

This manuscript is part of a larger research project that uses data from specific meta-analyses (see subsections below). The dataset gathered and analysed in the present study was also used to conduct a secondary meta-analysis [59]. Our methodology, summarized in the electronic supplementary material, figure S1, was described in our pre-registration [60], and we adhered to it as much as possible (see changes in the electronic supplementary material, S1). We report author contributions using MeRIT guidelines [61] and the CRediT statement [62].

### (a) Reported dataset

A recent systematic map identified the existence of 152 meta-analyses on topics related to sexual selection [7], 59 of them focusing on questions associated with ‘pre-copulatory sexual traits’ (i.e. sexual signals) [7]. In November 2023, P.P. selected 44 meta-analyses from this set, specifically the ones examining the relationship between sexual signals and distinct conditions, fitness proxies or individual traits (hereby *proxies*; see the electronic supplementary material, table S1). This means that P.P. excluded 15 meta-analyses that only explored other relationships of sexual signals, such as with species recognition (e.g. [63]), biotic factors (e.g. [64]), abiotic conditions (e.g. [65]), or other factors. P.P. also included another meta-analysis examining the relationship between ornament expression and parasite load [18], which was published after searches were conducted in [7]. This resulted in a total of 45 eligible meta-analyses to fulfil our objectives [18–58,66–69].

P.P. gathered the data reported by these meta-analyses from their tables, appendices, supplementary files and occasionally from direct correspondence with their authors. We could not obtain data for four eligible meta-analyses as their data were unreported [67–69] or poorly described [66]. Altogether, the remaining 41 meta-analyses from which P.P. extracted data [18–58] yielded 6773 data points (see §2e).

P.P. filtered the collated dataset for most analyses conducted here (see §2e and the electronic supplementary material, S2). This was done because not all data collected from meta-analyses were relevant to objectives of other parts of the larger research project (e.g. meta-meta-analysis of sexual signals; see [60]). Following this filtering process, the number of data points in the collated dataset decreased to 5496.

### (b) Re-extractions

Many meta-analyses included in the collated dataset extracted data from the same primary studies. More specifically, after filtering, P.P. detected that a quarter of primary studies in the dataset (318 out of 1272) were used as sources by at least two different meta-analyses (hereby *duplicates*; electronic supplementary material, figure S1). P.P. selected a subset of these duplicates for data re-extraction to reduce sampling effort (generating a greater sample size of data points for each meta-analysis assessed), prioritizing certain primary studies (see details in the electronic supplementary material, S3). In total, this process produced a set of 249 primary studies for re-extraction. However, we could not access the full-text of three of these studies, so our sample of studies for re-extraction was reduced to 246 (hereby *verified* primary studies; electronic supplementary material, S4).

We extracted all data on the relationship between sexual signals and proxies from verified primary studies (see details in the electronic supplementary material, S2), blinded to which exact data points were extracted for each of the meta-analyses that included a given study. More specifically, P.P. extracted data from 59.3% of the selected primary studies, while S.N., Y.Y., A.M., R.C.M.R. and M.L., respectively, extracted data from other 15.5%, 7.7%, 6.5%, 5.7% and 5.3% studies. P.P. then cross-checked all data extractions done by other authors (40.7% of studies). Importantly, authors never re-extracted data from primary studies they participated in. However, we cannot rule out that we experienced some unconscious bias when dealing with the meta-analyses of co-authors. We extracted data required to calculate effect sizes from text, tables, supplementary material and figures (using the package *metaDigitise* 1.0.1 [70]). When primary studies reported similar results in various forms, we prioritized extractions in the following order: (i) raw data (calculating estimates directly) from sources other than figures; (ii) raw estimates (i.e. means and correlation coefficients) from sources other than figures; (iii) raw data or raw estimates from figures; and (iv) other estimates (e.g. *t*, *β*, *χ*^2^) regardless of their origin. Nonetheless, we first prioritized data sources that showed more details. For instance, if a correlation was given for all individuals in text but a scatter plot showed the same data with dots separated by age or sex, we collected data from the latter. In total, we extracted 5703 valid data points. We note that we do not claim that our re-extracted data points are more or less correct than the data originally reported by meta-analyses, yet we do expect them to converge, meaning that mismatches should be taken seriously given our transparent procedures.

### (c) Matching reported data with re-extracted data

P.P. carefully examined the inclusion criteria reported in meta-analyses to verify which re-extracted data points should have been included by them. However, P.P. found several issues with these inclusion criteria. First, the proxies and sexual signals that meta-analyses included were often vague or ambiguous. For instance, Dougherty [19] was interested in behavioural sexual signals but their dataset also included extended phenotypes (e.g. domes built by crabs). Even though these extended phenotypes can be considered behavioural products, it was unclear whether other similar structures (e.g. bowers, ornamented nests) were deemed valid for inclusion by Dougherty [19]. Second, we detected apparent inclusion criteria patterns in datasets of some meta-analyses’ that were not mentioned in text. For example, Nakagawa *et al*. [40] stated that they included data on reproductive success but the only reproductive success measure in their dataset was the number of fledglings, even though the studies they used for effect size extraction also contained other measures (e.g. number of eggs, number of hatchlings). Third, some meta-analyses outwardly contradicted their own information. For example, Weaver *et al*. [56] stated that they included standardized colour metrics (hue, chroma or composite measures of those) for carotenoid-based colours in adult birds, describing specific proxies in their table 2. Yet, they seemed to have included data points in which (i) the sexual signal was the size of a colourful plumage (e.g. patch size), (ii) individuals were juveniles (including when data points were separated by age), and (iii) proxies other than the ones reported in text were used (e.g. offspring size). We summarized all meta-analyses’ originally reported inclusion criteria, the ambiguities, omissions and contradictions we detected in them, and how we dealt with these issues for matching purposes in the electronic supplementary material, S5.

We then attempted to match data points reported in meta-analyses with the ones we re-extracted from primary sources. P.P. mainly used the description of sexual signals and proxies of each data point to match them with re-extracted data. When multiple data points from the same primary study had similar descriptions, we also used sample size and other additional information (e.g. statistics reported, if given) for matching purposes.

There were three possibilities for each matching attempt. First, when both original and re-extracted data points had a similar description, P.P. linked them by labelling the latter with the identity (ID) of the former (i.e. successfully matched them). Yet, it was common to find multiple data points in our re-extracted dataset that matched one or many data points from the originally reported dataset (or vice-versa), so this matching was not necessarily exact (see examples in the electronic supplementary material, S5). Second, there were cases in which we could not find original data points with an equivalent description to relevant re-extracted ones. We assumed that these data were missed or undetected by meta-analyses’ authors. This allowed us to obtain two aspects related to reliability: (i) the number of relevant data points that were absent in meta-analyses’ datasets despite being present in primary studies reported as sources (hereby *missing data*); and (ii) the number of primary studies that contained relevant data points that should have been used as sources but were not (hereby *undetected studies*). To clarify the latter, consider a hypothetical meta-analysis that investigated the relationship between X and Y, reporting data from 10 primary papers. We then noticed two other studies containing relevant data (relationship between X and Y) that were used as data sources by other meta-analyses in our dataset but not the hypothetical one, even though the data from these two primary studies fit the inclusion criteria reported by the hypothetical meta-analysis. We thus deem that the hypothetical meta-analysis failed to detect at least two relevant articles. Third, there were cases in which data points were shown in meta-analyses’ reported datasets but were absent in our re-extractions. We re-checked all of these latter cases: although some data points were not extracted by us because of our criteria (e.g. invalid proxy) or because we considered them repeated data, most of them could simply not be found in primary studies (see details in the electronic supplementary material, table S2). This could have happened if meta-analyses’ authors contacted primary authors and were thus able to obtain more data than what was shown in the primary articles (i.e.unpublished data). Alternatively, meta-analyses’ authors may have made mistakes during data extraction, even though we cannot ascertain when this was truly the case.

After P.P. finalized the matching process, A.M., M.L., R.C.M.R., S.N. and Y.Y. cross-checked matching decisions for five different primary studies each. This resulted in matching decisions for approximately 10% of all verified studies being cross-checked, partially assessing the reliability of our process.

### (d) Effect sizes

Originally reported data points were given as the following effect size types: Cohen’s *d* [22,24], logarithm of response ratio (logRR) [32], and Fisher’s *Zr* or correlation coefficients (*r*) (remaining meta-analytical studies). One meta-analysis in particular [42] only provided *p*-values and sample sizes, so P.P. calculated effect sizes from this information. We transformed effect sizes reported to *Zr* for all analyses except the replication of general results (see §2e). We also calculated *Zr* (along with its sampling variance) from all re-extracted data points. Additionally, effect sizes reported by Koch *et al*. [32] did not reflect the raw data that they provided (means and standard deviations), so P.P. re-calculated their effect sizes. All equations for calculation and conversion of effect sizes are given in the electronic supplementary material, S7, while the direction rationale applied to effect sizes is detailed in the electronic supplementary material, S8.

### (e) Analyses

First, we attempted to replicate meta-analyses’ reported mean effect sizes using their original datasets. To do so, we conducted a meta-analytical model for each meta-analytical study using all of their reported effect sizes together (i.e. global model, *sensu* [7]). However, for meta-analytical studies that only performed subgroup analyses, we only re-analysed the largest or first reported subgroup. For instance, Nolazco *et al*. [41] only analysed the relationship between plumage colour and proxies for each sex separately, so we re-analysed only their data related to females. Effect sizes for these replication analyses were of the same type as results reported by meta-analyses (see §2d). Meta-analytical models for all of our analyses contained multiple random factors (see the end of this section) but, for this replication analysis, we also included an additional random factor if provided by authors. For example, both Robinson & Creanza [46] and Sánchez-Tójar *et al*. [49] used population ID as an additional random factor in their models. Yet, we were unable to include these additional random factors in meta-analytic models when these variables were not provided by authors with the rest of the data (e.g. experiment ID in [19] and population ID in [50]) or when they were redundant (population ID was different for every source in [20]). Moreover, two meta-analytical studies from our collated dataset were excluded from this specific analysis: Parker & Ligon [42] did not provide a confidence interval (CI) for their mean effect size and only data points of interest were extracted from Thornhill *et al*. [55] (i.e. their dataset was not fully extracted). We also tested for signs of publication bias (small-study effect) in re-analysed datasets by adding the inverse of the effective sample size as a moderator in meta-analytical models (alternative Egger’s regression) [71].

Second, we attempted to replicate part of individual effect sizes reported in meta-analyses. To do so, we used linear models to compare effect sizes originally reported by meta-analyses with the effect sizes we re-extracted from primary studies. For this analysis, we only used exactly matched data, in which each originally reported data point was matched to a single re-extracted data point (see the electronic supplementary material, S6). In these linear models, the intercept was forced to be zero, originally reported effect sizes were the response variable, and re-extracted effect sizes were the predictor variable. We evaluated whether the 95% CI of the estimated slope in each linear model included the value 1, which would represent that reported and re-extracted effect sizes are very similar. We complemented this analysis by computing the repeatability of individual effect sizes, i.e. the proportion of the variance from their origin (reported versus re-extracted) from the total variance observed. We could not compare originally reported effect sizes with re-extracted effect sizes from 11 meta-analyses because they contained less than six exactly matched data points (the arbitrary threshold we established for this analysis), so we only reported results related to the remaining 30 meta-analyses in our dataset.

Third, we assessed meta-analyses’ reproducibility by comparing the results of four slightly distinct meta-analytical models for each meta-analytical study. In model 1, we analysed the filtered dataset, which contained only reported effect sizes. In model 2, we used the same data as in model 1, but we replaced reported effect sizes that came from verified studies with their equivalent re-extracted effect sizes (i.e. the ones that matched in description). In model 3, we used the same data as in model 2, but we also added other relevant re-extracted data points from verified studies that were originally missed by meta-analyses’ authors (i.e. missed data). In model 4, we used the same data as in model 3, but we also added all relevant effect sizes from undetected studies. Differently from our analysis attempting to replicate mean effect sizes (first described in this section), we conducted global models for all meta-analytical studies, even those that had done only subgroup analyses. We note that the datasets used for most meta-analyses remained similar across the four models described above because we did not re-extract data from all primary studies reported as sources (electronic supplementary material, figure S2). For example, Dougherty [19] extracted data from 197 primary studies, but we verified only 15 of these studies. This means that results from these distinct models were unlikely to change for meta-analyses with proportionally few verified sources. Thus, we additionally conducted the same four models described above using only data from verified studies (instead of using data from both verified and unverified studies) for 15 meta-analyses with at least 15 verified sources. Furthermore, we also evaluated whether the inclusion of missing data and undetected studies changed the generalizability of results by examining the *σ* (a measure of heterogeneity) from each random factor in meta-analytical models.

When applicable, we compared estimates from meta-analytic models both qualitatively and quantitatively. We first ascertained whether an estimate was positive, negative, or not different from zero (if its 95% CI overlapped zero), so that estimates with distinct classifications represented a qualitative difference. By contrast, a quantitative difference occurred when the absolute difference between two estimates produced a *z*-score of more than 1.96 (i.e. statistically significant, two-tailed *α* = 0.05), calculated as:


$$z=\frac{X_{reported}-X_{re-analysed}}{\sqrt{se_{reported}^{2}+se_{re-analysed}^{2}-2rse_{reported}se_{re-analysed}}},$$

where *X* represents the estimated mean effect size, se represents its standard error, and *r* represents the correlation coefficient between these two groups (set as 0.8 for all main results as we assume this correlation is high but not perfect; but see the electronic supplementary material, figures S5–S7 for results using alternative methods). Although we showed all mean effect sizes generated in the last set of analyses, where we conducted up to four models per meta-analysis, we only discuss comparisons between the first and last models for simplicity.

We conducted all analyses described above in R 4.4.0 [72]. Multilevel meta-analytical models were fitted using the *rma.rv* function from the package *metafor* 4.6−0 [73]. All meta-analytical models fitted contained primary study ID as a random factor. Species ID (non-phylogenetic effect) and phylogenetic relatedness were also added as random factors in meta-analytical models to analyse data from meta-analytical articles that involved multiple species [74]. However, we removed phylogenetic relatedness from meta-analytical models related to certain articles [22,28,35,36], otherwise some of them would not converge. Phylogenetic trees were built using the packages *ape* 5.8 [75] and *rotl* 3.1.0 [76]. We conducted repeatability analyses using the package *rptR* 0.9.22 [77]. Unless stated otherwise, estimates reported in the manuscript represent mean ± s.e.

## 3. Results

### (a) Reproducibility of mean effect sizes

**Figure 1.**
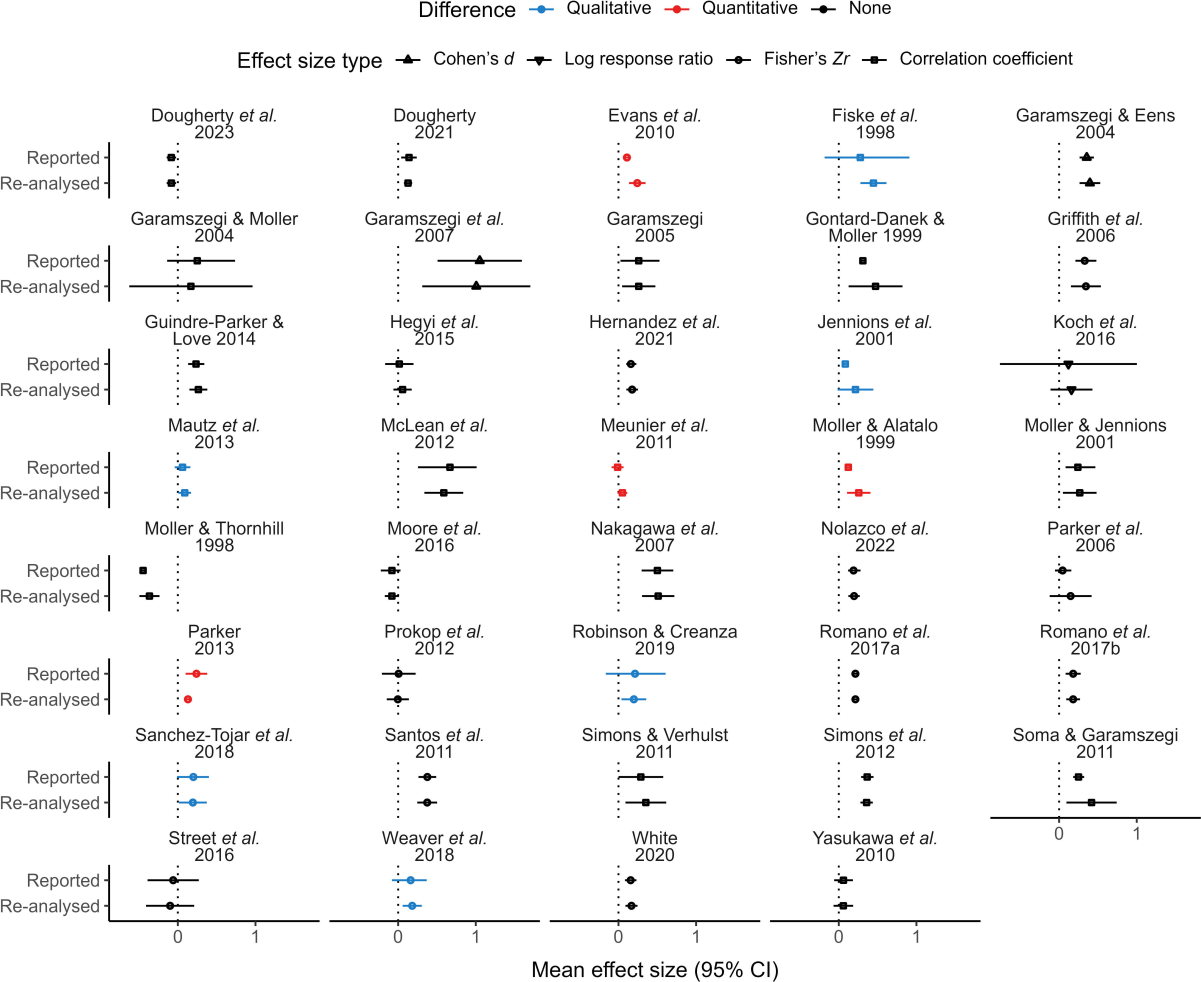
Originally reported and re-analysed mean effect sizes of 39 meta-analyses. Qualitative differences represent a change in interpretation between pairs (positive versus not different from zero or vice-versa), while quantitative differences represent statistical differences (absolute *z*-score greater than 1.96).

**Figure 2.**
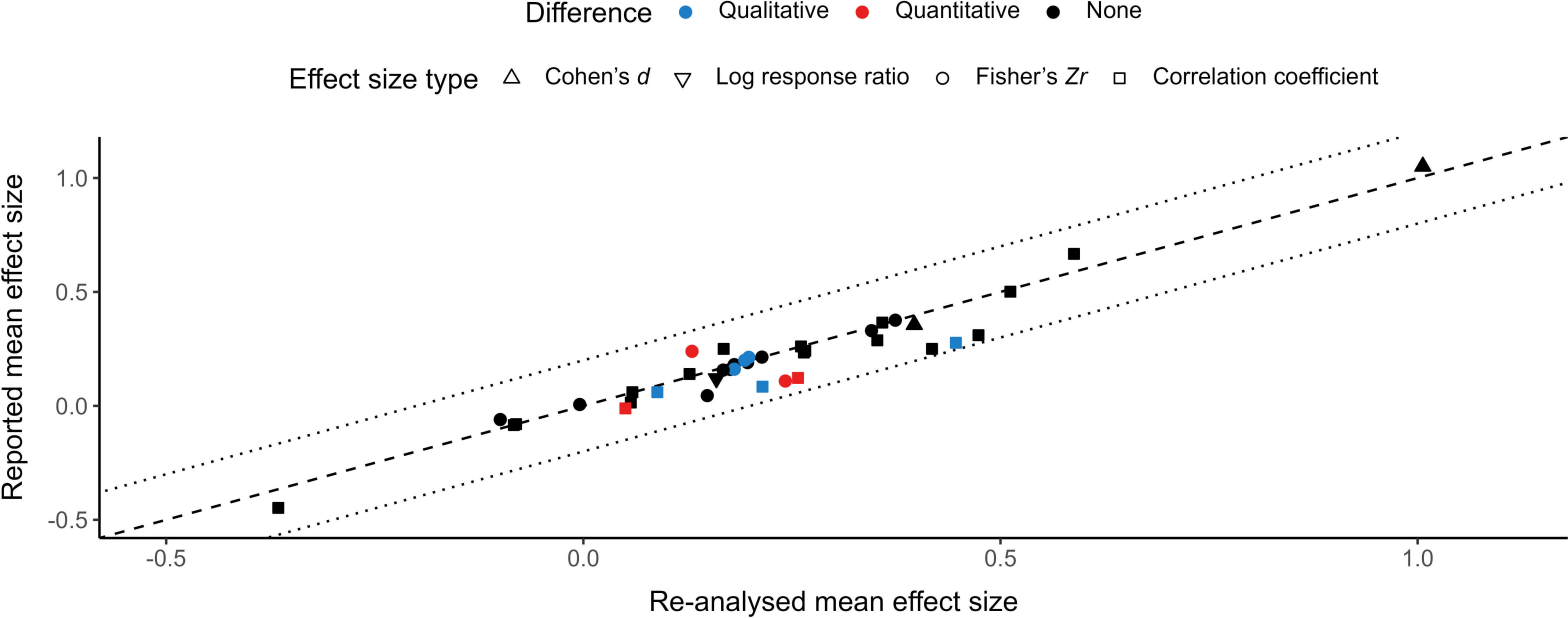
Comparison between mean effect sizes originally reported by meta-analyses and mean effect sizes resulting from the re-analysis of the reported dataset of the same meta-analyses. The dashed line highlights a perfect correspondence between variables, while dotted lines highlight a difference of 0.2 and −0.2 between reported and re-analysed mean effect sizes.

We found qualitative differences in 15.4% (6 out of 39) of the comparisons between mean effect sizes reported by meta-analyses and mean effect sizes resulting from re-analyses of those meta-analyses’ reported datasets (figure 1). More specifically, five of six meta-analyses reported a mean effect size not different from zero, but its re-analysed counterpart was positive, while another meta-analysis reported a mean effect size not different from zero, but its re-analysed counterpart was negative. We also detected quantitative differences between originally reported and re-analysed mean effect sizes (i.e. surpassed *z*-score of 1.96) in approximately a tenth of comparisons made (4 out of 39), although none of them were qualitative differences (figure 1; but see the electronic supplementary material, figure S3). Despite this, the absolute difference in magnitude between originally reported and re-analysed mean effect sizes was always lower than 0.2, even for cases with detected qualitative or quantitative differences (figure 2). We also found that effect sizes were positively predicted by effective sample size (i.e. evidence of publication bias) in 23.1% (9 out of 39) of meta-analyses. Two-thirds of these meta-analyses (6 out of 9) diligently reported that they detected publication bias [27,28,30,31,43,53], while the remaining third did not assess publication bias whatsoever [21,24,58] (electronic supplementary material, table S3).

### (b) Reproducibility of individual effect sizes

**Figure 3.**
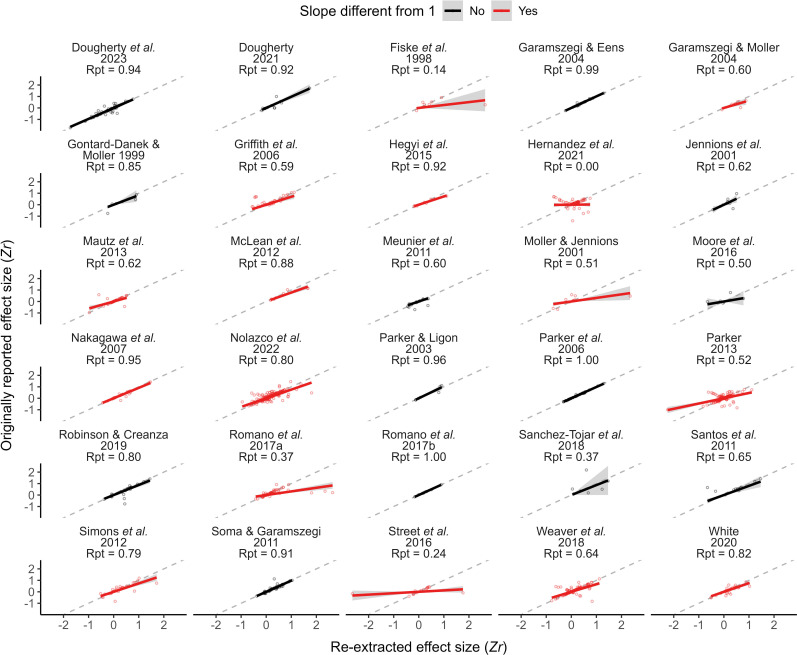
Relationship between effect sizes originally reported by meta-analyses and effect sizes re-extracted from primary studies. Solid lines represent a linear regression between reported and re-extracted effect sizes (with the intercept forced to zero), while shaded areas represent their 95% confidence interval. Red lines indicate slopes that significantly differ from 1. Dashed lines highlight a perfect relationship between reported and re-extracted effect sizes. ‘Rpt’ stands for repeatability of individual effect sizes.

We found that effect sizes originally reported by meta-analyses were, on average, statistically identical to their equivalent re-extracted ones in less than half (14 out of 30) of the meta-analyses evaluated (figure 3; electronic supplementary material, table S4). For the remaining meta-analyses, the slope from linear regressions between reported and re-extracted effect sizes was always lower than 1 (0.55 ± 0.07). While we observed, on average, a moderate repeatability of individual effect sizes (0.68 ± 0.05), meta-analyses whose re-extracted effect sizes we deemed statistically identical to the ones reported (i.e. slope not different from 1) showed higher repeatability in effect sizes than other meta-analyses (0.79 ± 0.05 versus 0.55 ± 0.07; electronic supplementary material, figure S4).

### (c) Missing data and undetected studies

**Figure 4.**
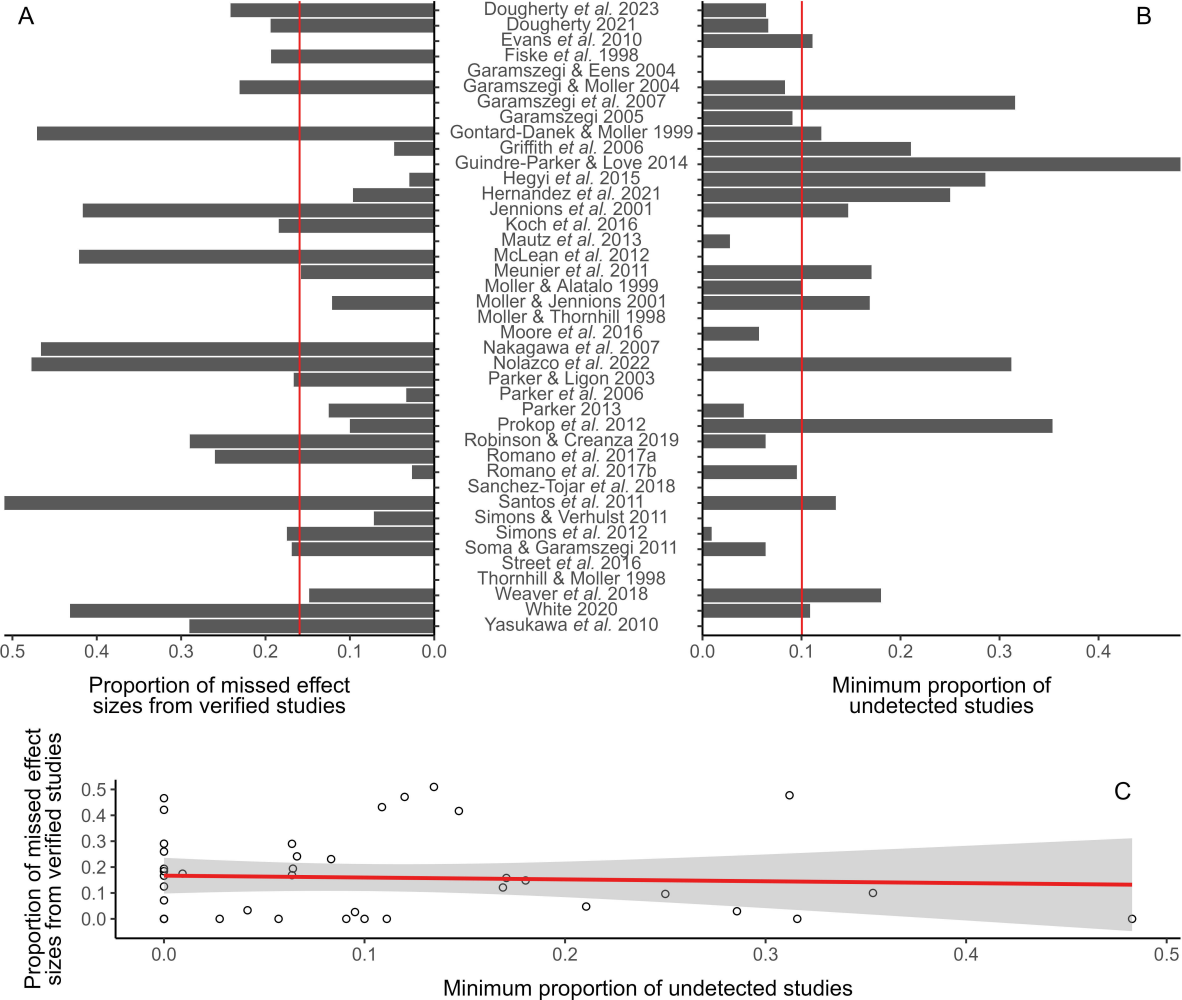
Proportion of missed effect sizes from all relevant effect sizes re-extracted from verified primary studies (A), proportion of undetected studies from the minimum number of primary studies that each meta-analysis should have included (B), and the relationship between these variables (C). Vertical red lines in panels A and B represent the average proportion of missed effect sizes from all relevant re-extracted effect sizes and the minimum proportion of undetected studies, respectively. The red line in panel C represents the fit of linear regression between variables with its 95% CI as the shaded area.

We found that meta-analyses missed (i.e. failed to extract and report), on average, 16 ± 2.6% of relevant effect sizes from primary studies they used as sources for data extraction (figure 4A). Additionally, we found that meta-analyses were unsuccessful in detecting, on average, at least 10% of primary studies they should have included as data sources (figure 4B). Furthermore, we found no association between the number of missing effect sizes and the minimum proportion of undetected studies across meta-analyses (figure 4C).

### (d) Replicability of results

**Figure 5.**
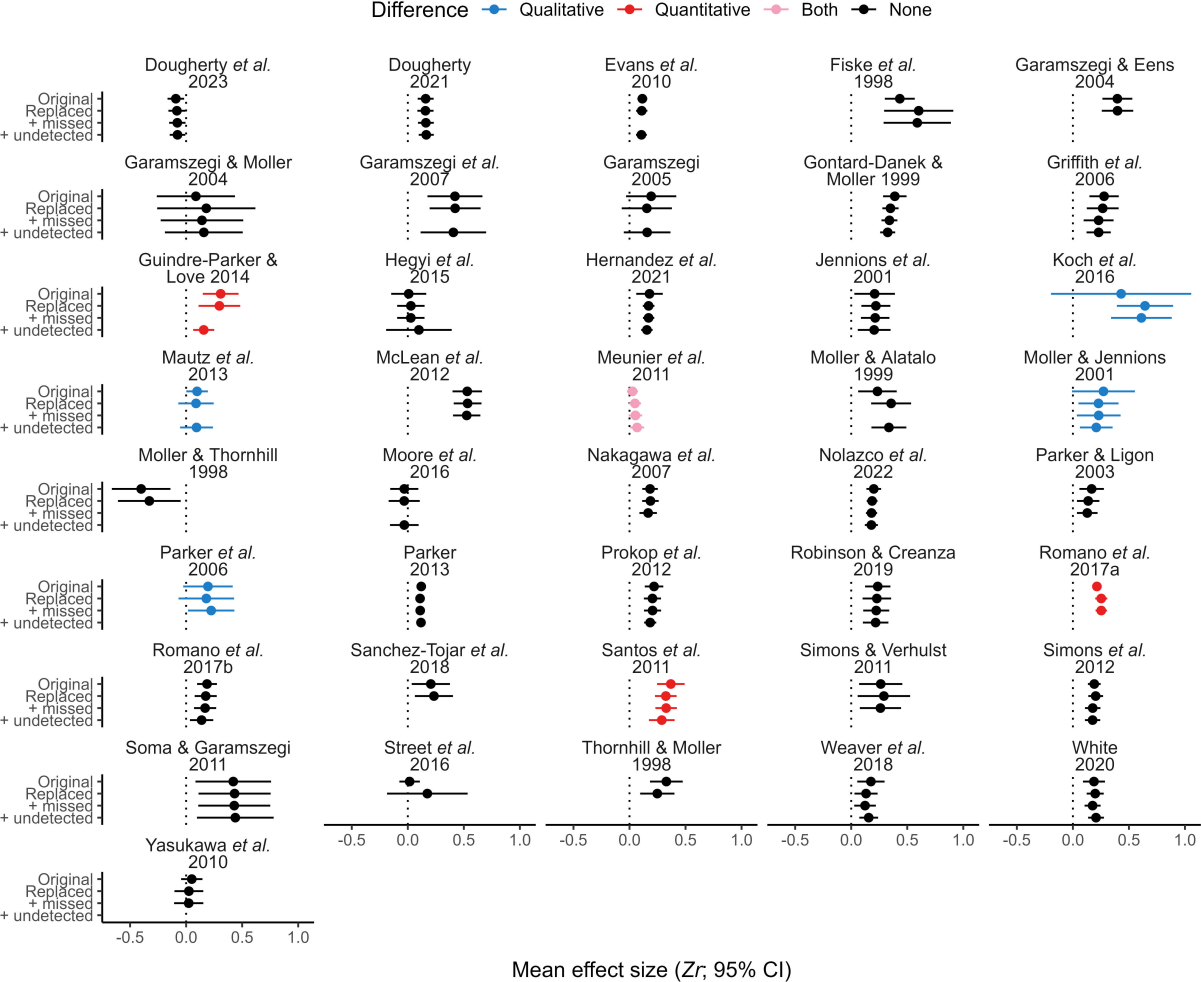
Mean effect sizes from up to four distinct meta-analytical models for each of 41 meta-analyses, using data from all primary studies (both verified and unverified by us, see details in text). Dotted lines highlight zero. Comparisons were made between the first and the last result shown within each subplot, with qualitative differences representing a change in interpretation (positive versus not different from zero or vice-versa) and quantitative differences representing statistical differences (absolute *z*-score greater than 1.96).

**Figure 6.**
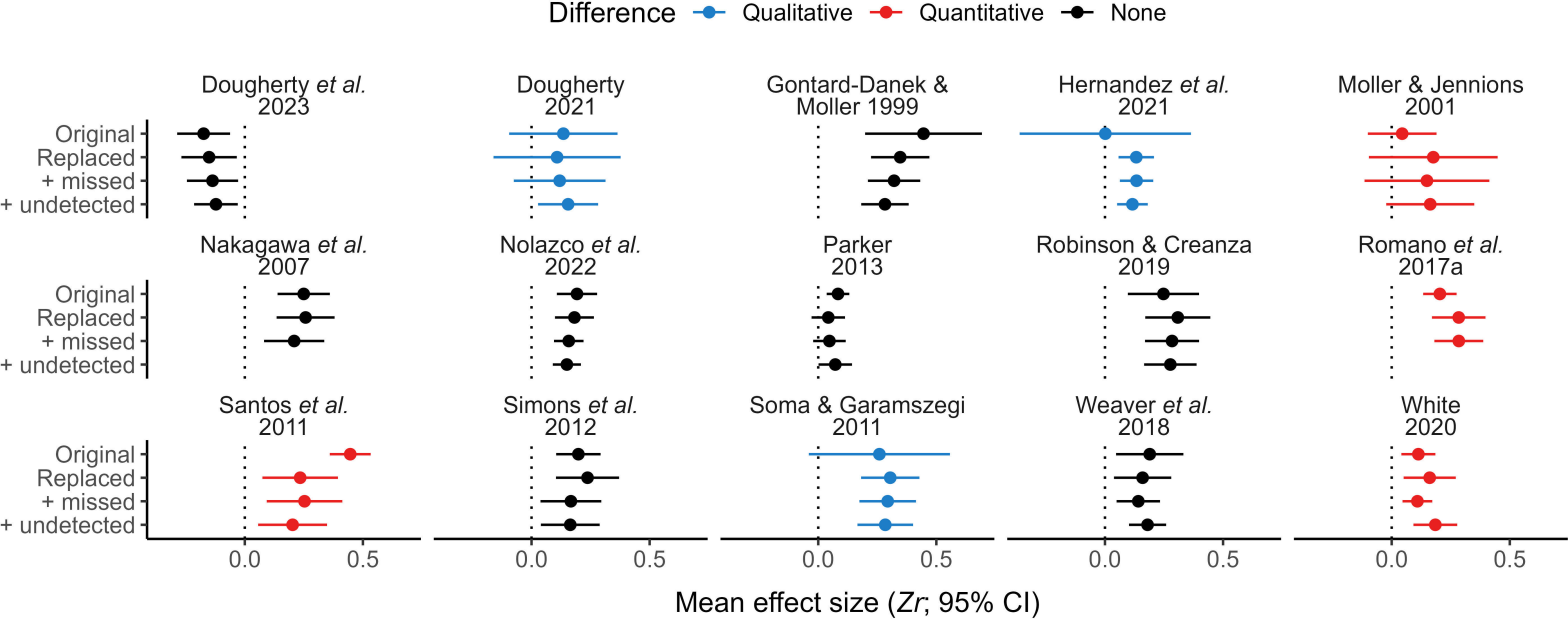
Mean effect sizes from up to four distinct meta-analytical models for each of 15 meta-analyses, using only data from primary studies that we verified (see details in text). Dotted lines highlight zero. Comparisons were made between the first and the last result shown within each subplot, with qualitative differences representing a change in interpretation (positive versus not different from zero or vice-versa) and quantitative differences representing statistical differences (absolute *z*-score > 1.96).

Using data from both verified and unverified primary studies (i.e. those for which we did or did not re-extract data, respectively), we found that mean effect sizes from analyses of originally reported datasets and those from analyses incorporating all relevant re-extracted data (matched, missed data, and undetected studies) were qualitatively and quantitatively distinct for 12.2% (5 out of 41) and 9.7% (4 out of 41) of meta-analyses, respectively (both types of differences occurred for one meta-analysis; figure 5; but see the electronic supplementary material, figure S5). Similar comparisons using only data from verified primary studies increased the occurrence of both qualitative differences (20%, 3 out of 15) and quantitative differences (26.7%, 4 out of 15; figure 6; but see the electronic supplementary material, figure S6). Nonetheless, differences in magnitude between mean effect sizes were often small (electronic supplementary material, figure S7). Furthermore, heterogeneity varied only slightly among fitted models (with perhaps one exception; electronic supplementary material, figure S8).

## 4. Discussion

In the first case study of the reliability of meta-analyses in ecology and evolution, we assessed a sample of meta-analyses that focused on the relationship between sexual signals and various measurements relevant to sexual selection. First, we were able to precisely replicate meta-analyses’ reported mean effect sizes in most but not all cases. Second, we found that effect sizes reported by meta-analyses and those we re-extracted did not overlap in multiple cases (i.e. the slope from linear regressions between reported and re-extracted effect sizes was statistically different from 1). Third, we observed that some relevant data, both from included and undetected studies, were missing from certain meta-analyses. Fourth, incorporating such missed relevant data into further analyses, in addition to replacing reported data from verified primary studies with re-extracted data, sometimes produced distinct meta-analytical results. However, the discrepancies we found tended to be small in magnitude and rarely generated distinct interpretations from the ones originally reported. Thus, we argue that the meta-analyses we evaluated are fairly reliable, although this is subjective to the degree of accuracy expected. Below we discuss the impacts of our findings and provide recommendations to further improve the reliability of meta-analyses in ecology and evolution.

Most of our results are based on comparisons between mean effect sizes (figures 1, 5 and 6) using categorical interpretations (i.e. negative, not different from zero or positive; indicating qualitative differences) and statistical calculations (indicating quantitative differences). We detected qualitative differences in only a handful of these comparisons, most of them resulting from differences in the width of mean effect sizes’ CIs rather than in their value. Similarly, we detected few quantitative differences when comparing mean effect sizes that ideally would have remained identical across analyses. Differences between re-analysed and reported mean effect sizes regarding the width of their CI can be explained by distinct analytical decisions from those originally conducted (e.g. inclusion of additional random factors). On the other hand, differences in mean effect size estimates could have also stemmed from errors made by the original authors in data analysis or result reporting. Regardless of the origin of the differences observed, these discrepancies are only relevant if they impact the interpretation of results from original meta-analyses. Ecology and evolution researchers rarely mention the magnitude of mean effect sizes from meta-analyses, focusing their attention on the existence or absence of effects or relationships [78]. This means that quantitative differences alone would probably be unimportant for most researchers (unless hypotheses rely on effect size comparisons, as in [13]), while qualitative differences would affect how results are perceived. However, even though interpreting results based on the inclusion of zero by CIs is a common practice, it can generate misleading conclusions [79]. For instance, the lower confidence bound for the mean effect size of Sánchez-Tójar *et al*. [49] was reported as −0.01 but was slightly above zero in all our analyses, while the magnitude of this study’s mean effect size was reported as 0.2 and ranged from 0.19 to 0.21 in our analyses (figures 2 and 5). Therefore, we conclude that results from Sánchez-Tójar *et al*. [49], as well as those from meta-analyses with similar minute discrepancies, should be deemed replicable despite being different to the ones we obtained when comparing the inclusion of zero by CIs. Importantly, statistical definitions of replicability and reproducibility are highly debatable, meaning that conclusions based on result comparisons become inevitably subjective to a certain degree [80,81].

Some effect sizes reported in meta-analyses were very different from the ones we re-extracted (figure 3). A portion of these discrepancies could be explained by the use of distinct effect size calculations, the extraction of data from distinct sources within primary studies (e.g. raw data from a figure versus statistics reported in text), or even the erroneous use of standard error instead of standard deviation in effect size calculations [82]. Yet, we noticed that numerous effect sizes reported were similar in value but opposite in direction to the ones we re-extracted. Although this did not strongly affect differences among estimated mean effect sizes, effect sizes with the wrong direction can be particularly dangerous as they should be more impactful than simply imprecise ones, at least for large effect sizes. Furthermore, errors in data extraction or effect size calculation can especially affect meta-regressions, which are often performed with subsets of meta-analytical datasets. This emphasizes the importance in meta-analyses of (i) establishing a coherent rationale to ascertain direction of effect sizes, (ii) reporting directionality decisions in detail, and (iii) remaining vigilant during data extraction. Additionally, we recommend cross-checking data extractions (i.e. independent verification of the data by someone who did not extract them) to increase the chances of spotting and correcting mistakes, including those related to the direction of effect sizes.

We observed that not all relevant data from verified primary studies were used by meta-analyses (figure 4). Although we cannot ascertain how or why meta-analyses’ authors missed relevant data from studies they extracted data from, the reason that some primary studies remained undetected might be more easily explained. While it is possible that meta-analyses could have employed suboptimal searches, primary studies commonly neglect to inform their entire scope and results in their title, abstract and keywords, which are used to retrieve and screen studies [83]. Thus, even though authors of meta-analyses should follow certain guidelines to build effective searches (e.g. [84]), meta-analysts may unfortunately miss relevant studies despite their best efforts. Conversely, authors of primary studies should be mindful of how search engines work, crafting their title, abstract and keywords to enhance the findability of their work [83,85].

The goal of our study was to evaluate the reliability of meta-analyses beyond transparency, yet our results might be affected by transparency issues found in the meta-analyses we assessed. First, matching reported and re-extracted data points proved to be a difficult task because meta-analyses usually lack details on their extracted data. For instance, the sexual signal and proxy for each data point were only vaguely described in most meta-analyses. Furthermore, no meta-analysis in our dataset reported the location of the extracted data within primary studies (e.g. which page, table and figure). Second, meta-analyses were often poorly transparent with their inclusion criteria. We tried to comprehend vague information, incorporate omitted criteria and ignore contradictions when matching re-extracted to reported datasets (see the electronic supplementary material, S5). Nonetheless, our decisions might have affected the amount of missing data and of undetected studies by each meta-analysis. Ultimately, this could have influenced our findings related to the reproducibility of mean effect sizes.

In addition to the recommendations we already mentioned (e.g. cross-checking of data extracted), we urge meta-analysts to provide all possible details on the data they collect. For instance, authors must clarify which exact measurements they sought instead of simply mentioning umbrella terms (e.g. condition-dependence by [28]). The location of the information is also crucial: readers should not have to examine datasets to find important details. Instead, summarized details should be in the manuscript or, less preferably, in the supplementary material, but always in a readable format (figures, simplified tables, in text, not in spreadsheets). Furthermore, we reiterate recommendations by Ivimey-Cook *et al*. [5], such as providing the within-text source for each data point extracted and the equations used to calculate effect sizes (along with assumptions and transformations used). We highlight that multiple factors can reduce the replicability of meta-analyses, such as large researcher degrees of freedom (i.e. decisions made by authors; *sensu* [86]), unclear or vague hypotheses, mistakes in data extraction, reporting errors or outright fraud. It is possible that meta-analyses in ecology and evolution can be particularly prone to suffer from some of these problems (e.g. large researcher degrees of freedom), given that researchers in this field explore broad questions with diverse taxa. Still, we argue that most of these issues can be improved, corrected, or at least detected by following our recommendations, summarized in the electronic supplementary material, table S5.

## Data Availability

All data and code used in this study are available at [87]. Supplementary material is available online [88].
